# Delivery of GM-CSF to Protect against Influenza Pneumonia

**DOI:** 10.1371/journal.pone.0124593

**Published:** 2015-04-29

**Authors:** Renuka Subramaniam, Zachary Hillberry, Han Chen, Yan Feng, Kalyn Fletcher, Pierre Neuenschwander, Homayoun Shams

**Affiliations:** 1 Center for Pulmonary and Infectious Diseases Control (CPIDC), The University of Texas Health Science Center at Tyler, 11937 U.S. Highway 271, Tyler, TX, United States of America; 2 Biomedical Research, The University of Texas Health Science Center at Tyler, U.S. Highway 271, Tyler, TX, USA; Indiana University, UNITED STATES

## Abstract

**Background:**

Since adaptive immunity is thought to be central to immunity against influenza A virus (IAV) pneumonias, preventive strategies have focused primarily on vaccines. However, vaccine efficacy has been variable, in part because of antigenic shift and drift in circulating influenza viruses. Recent studies have highlighted the importance of innate immunity in protecting against influenza.

**Methods:**

Granulocyte-macrophage colony stimulating factor (GM-CSF) contributes to maturation of mononuclear phagocytes, enhancing their capacity for phagocytosis and cytokine production.

**Results:**

Overexpression of granulocyte macrophage-colony stimulating factor (GM-CSF) in the lung of transgenic mice provides remarkable protection against IAV, which depends on alveolar macrophages (AM). In this study, we report that pulmonary delivery of GM-CSF to wild type young and aged mice abrogated mortality from IAV.

**Conclusion:**

We also demonstrate that protection is species specific and human GM-CSF do not protect the mice nor stimulates mouse immunity. We also show that IAV-induced lung injury is the culprit for side-effects of GM-CSF in treating mice after IAV infection, and introduce a novel strategy to deliver the GM-CSF to and retain it in the alveolar space even after IAV infection.

## Introduction

Seasonal influenza is one of the most important community acquired pneumonias in the U. S., causing 36,000 deaths and 200,000 hospitalizations annually (http://www.cdc.gov/flu/about/disease/index.htm). Influenza pandemics are also lethal and have killed up to 50 million people [1], and the recent swine influenza pandemic and the current avian H7N9 epidemic in Shanghai highlight this threat. Conventional immunization strategies have been the main focus of prevention that hinge on vaccines that require known antigens or live attenuated vaccine strains. Due to the specificity of conventional vaccines, the host will only be protected against the pathogen/pathogens for which the vaccine has been developed. Despite the many benefits of conventional vaccines that have saved millions of lives, this approach is neither designed for, nor capable of protecting the population against emerging infectious agents to which there is no vaccine available, and to which population have no prior exposure or immunity. Therefore, utilizing novel approaches that can effectively protect against emerging infectious diseases are of paramount importance.

Boosting innate immunity is a novel concept that can increase host defense against a range of pathogens, particularly those to which the population has no immunity nor there is any vaccine available. Since general stimulation of innate immunity may cause unwanted side effects, our focus has been on targeting local innate immunity elements in the lung. Pulmonary tract is one of the main routes of entry and transmission for many virulent pathogens such as influenza A virus, and a lethal route for select agents such as *Bacillus anthracis*, *Francisella tularensis* and *Yersinia pestis*, for which no vaccine or effective treatment is available.

We and others have shown that expression of granulocyte macrophage-colony stimulating factor (GM-CSF) in the lung provides remarkable protection against seasonal influenza (H3N2), PR8 (H1N1), pandemic flu (H1N1) and secondary bacterial pneumonia due to *Staphylococcus aureus* after influenza infection [2–6]. In these studies, the mortality from influenza pneumonia and secondary bacterial pneumonia infection in the transgenic mice that overexpress GM-CSF only in the lung was 0% compared to 100% in wild-type mice [2] and alveolar macrophages (AMs) were the major mediators of SPC-GM mice resistance that overexpress GM-CSF only in the lungs under the control of the human SP-C promoter [2–4]. In this report we expand our findings towards protecting wild type mice by pulmonary delivery of GM-CSF to the alveolar space to boost local innate immunity in the lungs prior to and post infection with influenza virus.

Delivery of high doses of GM-CSF (~100X of systemic dose) to the lungs is needed to be able to activate AMs. High concentrations of GM-CSF in the alveolar space are well tolerated as lungs are naturally semi-permeable to therapeutic proteins [7,8]. However, pulmonary infections such as influenza disrupt the barrier integrity of the lung and results in movement of fluid and macromolecules into and from alveoli that can result in reduced concentration of GM-CSF in alveolar space that will not be sufficient to activate AMs, and increased concentration of systemic GM-CSF that can cause toxicity and may even drain immune cells from alveolar space.

GM-CSF is associated with inflammation and asthma in some experimental systems [9–11]. However, high GM-CSF levels in healthy lungs show no ill effects, and aerosolized GM-CSF has not caused any toxicity in patients with acute respiratory distress syndrome, alveolar proteinosis or in GM-CSF-deficient mice [12–15]. More importantly, high local concentrations of GM-CSF in the alveolar space are prerequisite for its beneficial effects [15] and systemic GM-CSF delivery (intravenous infusion) in patients with sepsis-induced lung injury failed to improve the outcome [16]. This suggests that a direct route of delivery of GM-CSF to the alveolar space is required. Here, we show that influenza-induced lung injury facilitates the escape of intranasally delivered GM-CSF from the alveolar space. We introduce novel strategy to facilitate retention of GM-CSF in the alveolar space to sustain the high concentration of GM-CSF that is required for AM activation, and reduce the potential for systemic toxicity of GM-CSF delivered to respiratory tract. This strategy seems feasible for protecting population against infectious agents such as pandemic influenza and bioterrorism agents that pose a risk to national security due to their easy dissemination, unknown sources, and high mortality rates.

## Materials and Methods

### Mice

Eight 10-week-old wild type C57BL/6 and Balb/c mice were purchased from Jackson laboratory and 20-months old C57BL/6 mice were purchased from National Institute of Aging. All mice were maintained in the vivarium at the University of Texas Health Science Center at Tyler.

### Ethics Statement

All experimental animal protocols were approved by the Institutional Animal Care and Use Committee (IACUC) at the University of Texas Health Science Center at Tyler. Mice were observed at least once daily by authorized personnel for signs of distress, weight loss, ruffled fur, hunched back posture and lack of movement. Mice that lost more than 20% of their bodyweight were evaluated by veterinary staff. Experimental mice were euthanized by Beuthanasia or Euthasol which were administered intraperitoneally (ip) at a dosage of 5ul/g body weight. Any intranasal treatment was performed under deep anesthesia with Ketamine HCL (100mg/kg) and Xylazine (8.5 mg/kg) ip. Experimental mice were euthanized if lost more than 30% of their weight and were distressed with ruffled fur, hunched back posture and lack of movement.

### Influenza A virus infection

The mouse-adapted influenza virus A/Puerto Rico/8/34 (PR8) (H1N1) strain was used in all experiments. Mice were intranasally inoculated with 50 μl of PBS containing the H1N1 PR8 strain under light general anesthesia with a combination of Ketamine/Xylazine. All infected mice were monitored for weight loss and mortality on a daily basis.

### Intranasal treatment with GM-CSF

Mice were treated intranasally daily with recombinant murine GM-CSF (Invitrogen) or recombinant human GM-CSF (LEUKINE) for 7 days, prior to infection with PR8 H1N1 influenza. Seven days treatment regimen has been shown to be optimal in our previous publication [2].

### Mouse bone marrow proliferation assay

Mouse bone marrow cells from C57BL/6 mice were incubated with plain or conjugated mouse GM-CSF. Ninety six hours later, WST-1 reagent (Roche, #05-015-944-001) was added (10 μl/well) to the plate and incubated for 4 hrs and the absorbance was measured. WST-1 is a colorimetric assay to assess the number of viable cells by the cleavage of tetrazolium salts added to the culture medium.

### Measuring TNF-α, MCP-1 and amphiregulin levels

Mice were treated with human or mouse GM–CSF or PBS as described above. After seven days of treatment, lungs and bronchoalveolar lavage (BAL) fluids were collected and stored at -80°C. Levels of TNFα and Amphiregulin in supernatants of BAL fluid and level of MCP-1 in supernatants of lung homogenate were measured by enzyme-linked immunoassay kit (TNFα and MCP-1 from eBiosciences, CA; and Amphiregulin kit from R&D systems, MN).

### Mouse alveolar macrophages proliferation assay

Mice were treated with either mouse or human GM-CSF as described in 2.3 for 7 days. Broncho alveolar lavage was collected on day 8 and the number of alveolar macrophages was counted for each individual mouse. We stained Bronchoalveolar lavage cells with anti-F4/80 to identify AM, and found that over 95 percent of BAL cells were AMs.

### Measuring PU.1 in the lungs

Lungs from the mice treated with either mouse or human GM-CSF as described in 2.3 were collected and lung homogenates were prepared. Thirty μg of lung homogenate from mGM-CSF-treated mice, hGM-CSF-treated mice and un-treated control mice were electrophoresed through 4–15% SDS polyacrylamide gel. Samples were transferred onto the PVDF membrane and developed using rat anti-mouse PU.1/Spi-1 antibody (R &D systems, MN). Density of the bands were quantified using Image lab software version 5.0 (Bio-Rad, CA).

### Using Quantum Dots (Qdots) to detect permeability of lung vasculature in IAV-infected mice

Commercially available Qdots are between 10–20 nm in diameter (similar in size to human serum albumin for comparison; ~8–10 nm). Thus, Qdots were used as crude molecular rulers to measure the pore size of the permeable lung vasculature after infection. The Qdots used in this experiment were either 13 nm (Qdot 525) or 20 nm (Qdot 625) in diameter and emitted light at 525 nm and 625 nm wavelength, respectively, allowing clear discrimination of particle size from various samples using a standard fluorimeter. Several initial experiments were conducted to optimize the Qdot dose. Intravenous injection of 50 μl of each Qdot was sufficient for detecting Qdots up to 4 h after intravenous administration in urine, lung homogenate and BAL.

### Conjugation of mouse GM-CSF to Qdot

Qdots (625 nm) were activated with 20 μM EDC/NHS for 15 min at room temperature. The activation reactions were terminated using DTT, and mGM-CSF was then added (final concentration of 0.5 mg/ml) and amine coupling allowed to proceed for 2 h at room temperature before quenching with 5 mM ethanolamine. Conjugated mGM-CSF to Qdot samples were filtered and concentrated using a 30,000 MWCO Centricon to separate conjugated material from non-conjugated mGM-CSF in solution. Qdot-mGM-CSF were recovered and subjected to protein concentration analysis by ELISA and determination of biological activity by cell proliferation assay. The efficiency of the conjugation reactions was determined by the ratio of conjugated to non-conjugated GM-CSF after centrifugation.

### CT-scanning of IAV-infected mice

Mice were infected with IAV and imaged post infection using a GE eXplore Locus micro CT scan (X-ray tube voltage of 80 kVp, tube current of 450 μA). The exposure time was 90 ms per projection and the gantry was rotated over 200 degrees while 200 two dimensional projections were taken.

### Statistics

GraphPad Prism software was used for statistical analysis. Survival curves were analyzed by chi-square test. Comparisons between two groups were calculated by unpaired Student’s t test and p<0.05 was considered statistically significant. All measures of variance are presented as SEMs.

## Results

### Mouse GM-CSF protects mice against lethal influenza infection

**Fig 1:**
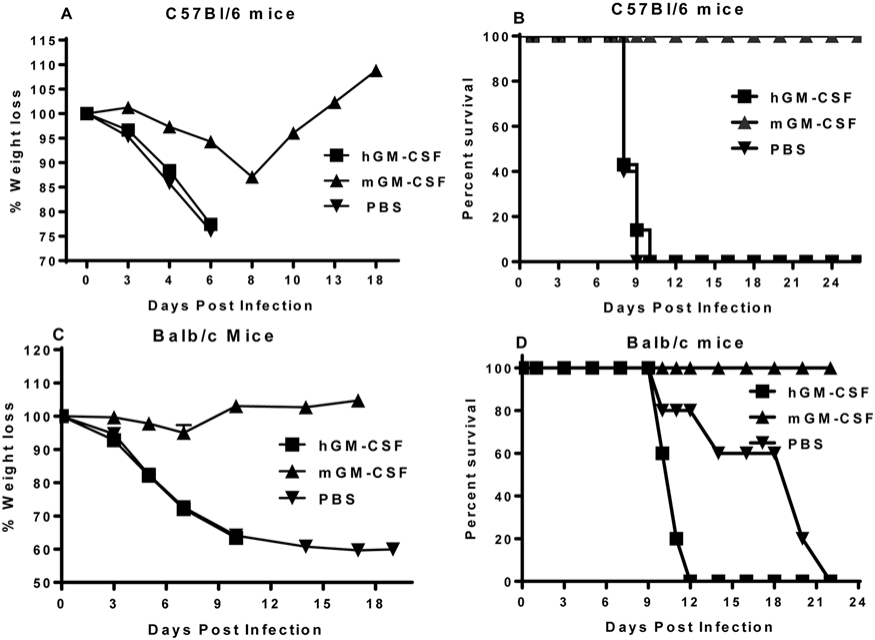
Pulmonary GM-CSF protects mice against influenza infection. C57BL/6 (A and B) and Balb/c (C &D) mice were treated with either murine GM-CSF (mGM-CSF) or human GM-CSF (hGM-CSF) intranasally daily for one week, and a control group was treated with PBS. Mice were then infected with a lethal dose of mouse-adapted influenza A virus PR8. Infected mice were monitored and their weight loss (A and C) and mortality (B and D) were recorded daily. A representative of two to four independent experiments with similar results is shown. (n = 5–7 mice/group).

Biological activity of recombinant mouse and human GM-CSF (mGM-CSF and hGM-CSF, respectively) was evaluated with respect to protection against influenza virus. We treated C57BL/6 mice with either mGM-CSF or hGM-CSF intranasally daily for one week (1.34 μg/g body weight). A control group was treated with PBS. All mice were then infected with lethal dose (2 LD50) of mouse-adapted influenza A virus PR8. Infected mice were monitored daily and their weight loss and mortality were recorded. All C57Bl/6 mice treated with PBS or hGM-CSF and infected with PR8 lost more than 25% of their body weight whereas mGM-CSF treated mice showed less than 15% weight loss (Fig 1A). Next, we tested the protection of treated mice against lethal dose of 2 LD50 of PR8. All treated mice with PBS and hGM-CSF succumbed to infection 7–10 days after infection while all mGM-CSF treated group survived (Fig 1B).

To confirm our finding is not specific in C57Bl/6 genotype, we repeated the experiments carried out in Fig 1A and 1B using Balb/c mice. Balb/c mice were treated with either mGM-CSF, hGM-CSF or PBS as described above and were infected with a lethal dose of influenza A virus PR8 (2 LD50). All PBS- and hGM-CSF-treated Balb/c mice lost more than 30% of their body weight and died by 12–22 days after infection. However, mGM-CSF-treated Balb/c mice lost less than 15% body weight and all survived the infection (Fig 1C and 1D).

### Mouse GM-CSF increases the number of AMs in the lungs

**Fig 2:**
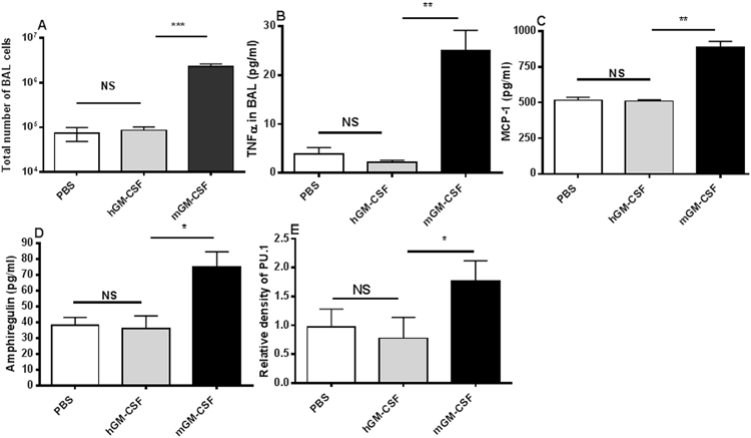
Effects of GM-CSF on alveolar macrophages, lung cytokines and growth factor. Wild type C57Bl/6 mice were treated intranasally daily with murine GM-CSF (mGM-CSF) or human GM-CSF (hGM-CSF) for 7 days. On day 8, mice were sacrificed and the number of alveolar macrophages in BAL (A), levels of TNFα (B), MCP-1 (C), amphiregulin (D) and expression of PU.1 (E) in the BAL/lungs were measured. Depicted data show mean of 3–5 animals per group ±SEM and a representative of two independent experiments. *p<0.01, **p<0.001, ***p<0.0001.

Next we evaluated the effects of mGM-CSF and hGM-CSF on total cell counts of bronchoalveolar lavage (BAL) that were over 95% alveolar macrophages and an indicator of in vivo immune cell proliferation in the alveolar space. Overexpression of mGM-CSF in the lungs in mice significantly increases the numbers of alveolar macrophages [7]. Wild type C57BL/6 mice were treated with either mGM-CSF, hGM-CSF or PBS intranasally daily for seven days (1.34 μg/g body weight). On day eight, mice were sacrificed and their lungs were lavaged. Mice treated with PBS and hGM-CSF had comparable number of AMs in their BAL whereas mGM-CSF-treated mice showed markedly increased number of AM and had approximately 27 times more AM than PBS- and hGM-CSF-treated mice (Fig 2A, P<0.0001).

### Effects of mGM-CSF on cytokines and growth factors in the lungs

This is a well-established fact that GM-CSF is a pleiotropic cytokine. Its effects on hematopoietic cells consist of stimulation, proliferation and differentiation. These functions are mediated either directly through binding to GM-CSF-receptor or indirectly through the network of numerous cytokines, chemokines and growth factors [17]. Hence, we measured concentrations of selected cytokines and a growth factor (amphiregulin) in lungs of mice treated with mGM-CSF, hGM-CSF or PBS. Naïve mice treated with mGM-CSF had high levels of tumor necrosis factor (TNF)-α and monocyte chemoattractant protein (MCP)-1, compared to hGM-CSF-treated and PBS-treated naïve mice (Fig 2B and 2C). In mGM-CSF-treated mice, TNF-α levels increased more than twelve times of PBS-treated baseline (24.9 ± 4.2 versus 3.95 ± 1.23, p<0.001) and more than six times of hGM-CSF-treated levels (24.9 ± 4.2 versus 2.1 ± 0.49, p<0.001). Next, we measured the levels of Amphiregulin that is an epidermal growth factor and has been shown to be increased in the lungs by GM-CSF [6]. In mice treated with mGM-CSF, amphiregulin levels also rose markedly compared to PBS-treated (75.2 ± 9.5 versus 36.0 ± 8.1, p<0.01, Fig 2D) and hGM-CSF-treated groups (75.2 ± 9.5 versus 38.2 ± 4.9, p<0.01, Fig 2D). To assess the effects of GM-CSF on transcription factor, we measured expression of ets family transcription factor PU.1 that regulates production and development of macrophages, B lymphocytes, neutrophils and T lymphocytes in the lungs of GM-CSF-treated mice [18]. Wild type C57Bl/6 mice treated with mGM-CSF had significantly higher expression of PU.1 in the lung compared to their counterparts that were treated with either hGM-CSF or PBS (Fig 2E).

### mGM-CSF protects aged mice against lethal influenza infection

**Fig 3:**
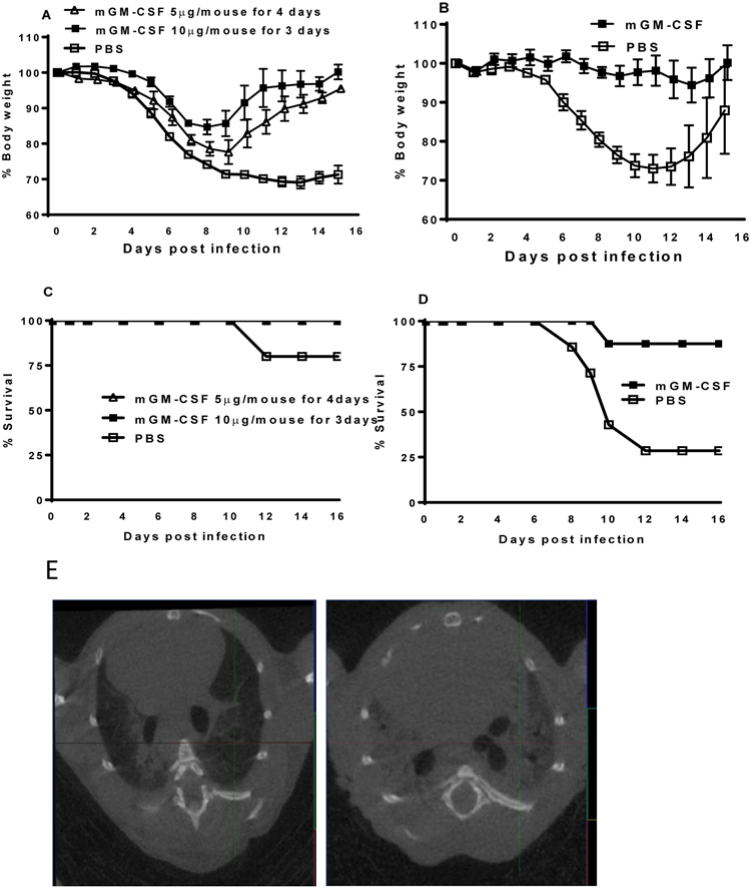
Effect of treating IAV-infected aged mice with GM-CSF. **(A&C)** Aged (20-months old) C57Bl/6 WT mice (5/group) were treated with PBS or two mGM-CSF regimens, and then infected with a sublethal dose (0.1 LD50) of IAV PR8, 24 hrs after the last treatment. **(B&D)** Aged C57Bl/6 mice were treated with 1.3 μg mGM-CSF/g body weight (~26μg/mouse) or PBS daily for 1 week, and infected 24 hrs later with a lethal dose of PR8 (0.5 LD50). Weight loss and mortality were recorded daily. **(E)** Anatomical changes in the lungs of mGM-CSF-treated **(E, left panel)** and PBS-treated **(E, right panel)** aged mice after infection with IAV. Mice were treated and infected, as in panel B. Ten days post-infection, CT scanning was carried out, using a GE eXplore Locus micro CT scan. Transverse sections of the thorax are depicted.

It is known that elderly individuals are at higher risk for complications from influenza, and vaccination is less effective in this population [19]. Thus, in order to test the efficacy of GM-CSF-based therapy and to establish a protective regimen in aged mice, we delivered different doses of GM-CSF intranasally to the lungs of 20-months old WT mice. All PBS-treated aged mice infected with a sub-lethal dose (0.1 LD50) of PR8 lost weight, which they did not regain, and 1 of the 5 mice died (Fig 3A and 3C). In contrast, animals pretreated with two different regimens of GM-CSF (either 5 μg/day for 4 days or 10 μg/day for 3 days) regained weight by 14 days post-infection (Fig 3A). The protective effect of intranasal mGM-CSF was even more dramatic when the aged mice treated with high dose of mGM-CSF (1.3 μg mGM-CSF/g body weight (~26μg/mouse) for 1 week) and the infectious dose of PR8 was increased to 0.5 LD50. Animals pretreated with high dose mGM-CSF showed nominal weight loss and 87.5% survival compared to 29% for PBS-pretreated mice (Fig 3B and 3D). Micro-CT scanning provides great technology to evaluate disease severity in live animals. We used Micro-CT scanning (GE eXplore Locus) to evaluate anatomic changes and disease severity in live animals. On day 10 after IAV infection, micro-CT scanning was performed on the mGM-CSF- and PBS-treated control mice. PBS-treated IAV-infected aged mice showed extensive consolidation, reduced aerated lung volumes and pulmonary ground-glass opacities, indicative of severe pneumonia and/or pulmonary edema whereas mGM-CSF-treated mice infected with IAV had clear and normal lungs, as shown in transverse sections (Fig 3E).

### Influenza induces lung injury

e have shown that pulmonary infections such as influenza induce lung injury and disrupt the barrier integrity of lung vasculature and results in increased albumin levels in the alveolar space [2]. On the other hand, GM-CSF will be clinically very useful if given to patients after the onset of influenza, but in our hands treating IAV-infected mice after 2 days post infection has mostly been fatal (data not shown).

**Fig 4:**
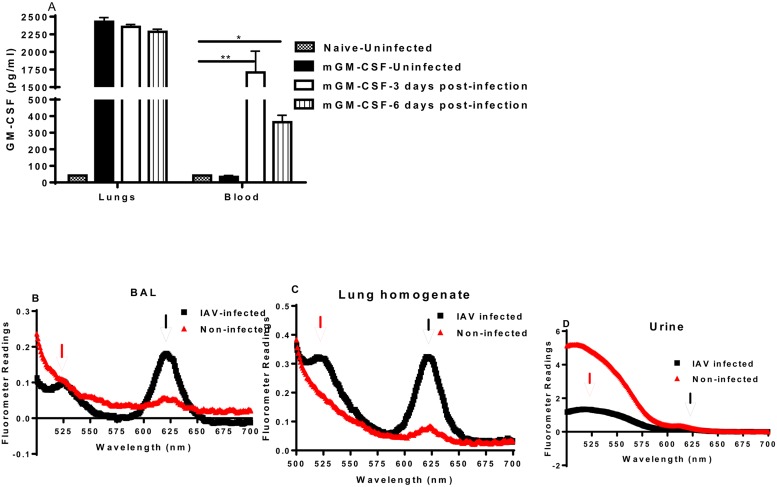
Lung injury in IAV-infected lungs facilitates movements of molecules from and to the alveolar space. **A)** Groups of wild type C57BL/6 mice were infected with 1.0 LD50 of PR8 and then treated intranasally with 12.5 μg of GM-CSF on days 0, 3, and 6 after IAV infection. Levels of GM-CSF are shown 3 hours after treatment. * p<0.001, **p<0.0001. n = 5. **B-D) The abundance of Q-Dots in BAL, lungs and urine**. Wild type C57Bl/6 mice were infected as in panel A and 7 days after infection, mixture of two different Qdots (13nm and 20nm) were given intravenously. Passage of different particle size through the lung vasculature and glomerular capillary filtration of naïve and IAV-infected mice are depicted. Representative of three independent experiments with similar results are shown from 3–5 mice/group. Samples from naïve mice were used as controls and showed no fluorescence. Red and black arrow indicates 13nm and 20 nm Qdots, respectively.

When WT mice were infected with 1.0 LD50 of PR8 and then treated intranasally with GM-CSF, there was a significant increase in the concentration of GM-CSF in the blood of PR8-infected mice only three hours after treatment with GM-CSF at 3 and 6 days post-infection indicating that lung injury induced by influenza infection induces a porous lung that facilitates the escape of GM-CSF from alveolar space (Fig 4A).

We next used a novel approach to confirm the lung injury of IAV-infected mice and crudely assess the pore size of the permeable IAV-injured lung vasculature/epithelium. The endothelial cell lining of the pulmonary vasculature forms a semi-permeable monolayer barrier between the blood and the interstitium of the lung. Pulmonary infections such as influenza disrupt the barrier’s integrity, resulting in facile movement of fluid and macromolecules into and out of alveoli. We used Qdot ITK carboxyl quantum dots that are fluorescent semiconductor-based nanocrystals (Life Technologies). These nanocrystals show a direct, predictable relationship between their physical size and the energy of the excitation, and consequently display a different wavelength of emitted fluorescence from different sizes. We used commercially available Qdots in this experiment that were either 13 nm (Qdot 525) or 20 nm (Qdot 625) in diameter and emitted light at 525 nm and 625 nm wavelength, respectively, using a standard fluorimeter. Qdots were administered intravenously into the tail vein of either naïve mice or IAV-infected mice seven days after infection. Samples were collected an hour later. Shortly after intravenous injection of Qdots, the 13-nm Qdots passed through glomerular capillaries of kidneys and were found in abundance in urine samples of naïve mice whereas the 20-nm Qdots did not (Fig 4D). Samples from naïve mice were used as controls and showed no fluorescence activity (data not shown). Interestingly, the concentrations of Qdots in the lungs and BAL samples of IAV-infected mice were much more than in the lungs and BAL samples of non-infected naïve mice, indicating that the lungs’ vasculature/epithelium of IAV-infected animals were more permeable to Qdots than those of naïve control miceCBA (Fig 4B and 4C).

### Conjugation of mGM-CSF to Qdot and biological Activity of Qdot-mGM-CSF

**Fig 5:**
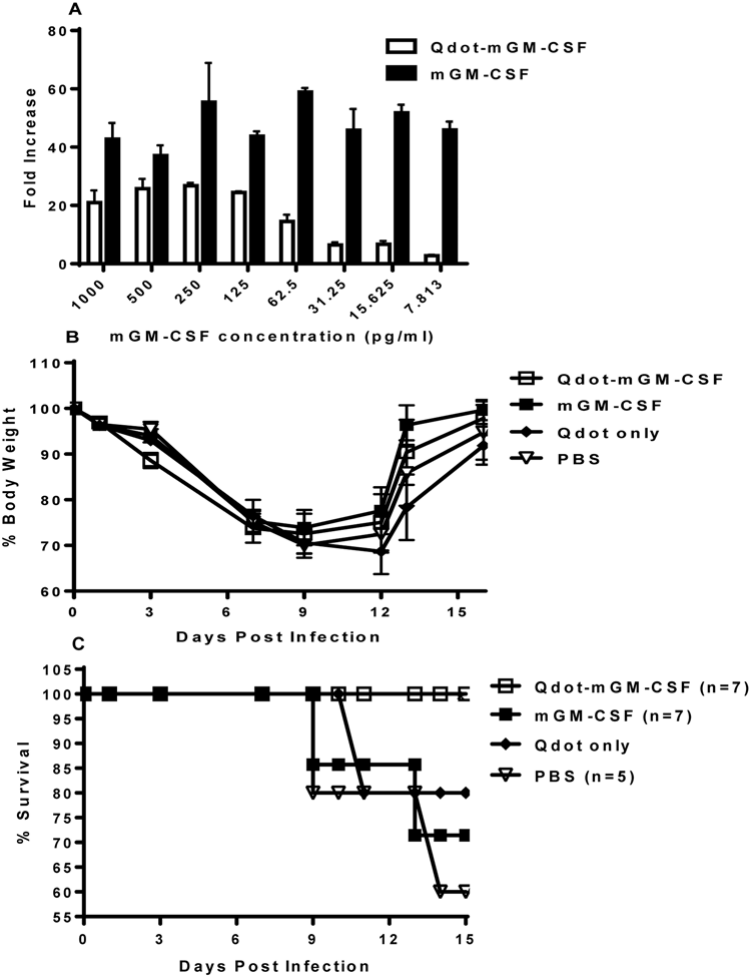
Biological activity of mGM-CSF after conjugation to Q-Dots. A. Bone marrow proliferation was used to measure biological activity of the Qdot-mGM-CSF. Different concentrations of plain mGM-CSF and QDots-mGM-CSF were prepared in a 96-well plate (final volume of 50ul/well), starting at 1μg/ml. Non-adherent bone marrow cells from C57/BL6 mice were suspended in minimum essential medium and added to each well (3x10^4^/well) and incubated at 37°C for 96 hrs. Proliferation was measured by using WST-1 reagent. **B-C**. Wild type C57/Bl6 mice were infected with PR8 IAV. Two groups of 7 IAV-infected mice were treated with 12.5 μg of either mGM-CSF or mGM-CSF conjugated to a 625nm fluorescent Q-dot (Qdot-GM-CSF), one group with Qdot only and the fourth group treated with PBS. All groups were treated twice after IAV infection. All mice were monitored daily and their weight loss (B) and mortality (C) were recorded. A representative of two experiments with similar results is shown. Error Bars represent mean ± SEM.

We decided to take a novel approach and conjugate mGM-CSF to Qdot particles to increase its size and facilitate retention of delivered GM-CSF in the alveolar space. Recombinant mGM-CSF was conjugated to Qdot 625 particles using standard amine-coupling chemistry [20]. Conjugation efficiency was estimated by ELISA analysis of the post-conjugation solution, obtained as the flow through of filtrated conjugated Qdot-mGM-CSF and determined to be 99.9% (data not shown). Then, we measured the biological activity of Qdot-mGM-CSF using mouse bone marrow proliferation assay. The biological activity of Qdot-mGM-CSF was slightly reduced when compared to a non-conjugated mGM-CSF (Fig 5A). However, the conjugated protein still exhibited proliferation of bone marrow cells more than 20-fold greater than a non-treated control at 125 ng/ml and was considered biologically active at that concentration.

### Treating IAV-infected mice with plain mGM-CSF and Qdot-mGM-CSF

In order to test the efficacy of Qdot-mGM-CSF in protecting IAV-infected mice, wild type C57BL/6 mice were infected with a sub-lethal dose (0.2 LD50) of influenza strain PR8 and then divided into four groups. IAV-infected mice were treated with PBS or 12.5 μg of either mGM-CSF, Qdot-mGM-CSF or Qdot only at 3 h and 24 h post-infection. There was no significant difference in the weight loss among the four groups (Fig 5B). However, all the mice treated with Qdot-mGM-CSF survived whereas two of five PBS-treated, one of Qdot only treated mice and two of seven GM-CSF-treated mice died. There was no statistical significance in the survival rate between mGM-CSF and PBS groups, but the group treated with Qdot-mGM-CSF showed better trend in survival compared to both mGM-CSF and PBS groups (Fig 5C), indicating the need for improvements in both conjugation and delivery.

## Discussion

GM-CSF is among the first cytokines identified, is a pleiotropic cytokine and its biological activity is mediated by binding to specific cell surface receptors [21]. GM-CSF binds to GM-CSF-specific receptors (GMR) and promotes proliferation, differentiation, and survival in myeloid precursors as well as inducing the effector functions of mature myeloid cells [22–24]. GMRs in humans comprise an α chain (hGMR-α) and a β subunit (hβc) that transduces signals and is shared with the interleukin-3 (IL-3) and IL-5 receptors [25]. In the murine system there are two β subunits, mβc, that are analogous to hβc and induced by mouse (m)GM-CSF, mIL-3 or mIL-5.

GM-CSF has been used by our lab and others to protect against influenza, secondary bacterial pneumonia, and acute lung injury [2–6,8,13–15,26–28]. In addition to the FDA approved application for GM-CSF for treating bone marrow suppression, these recent studies have generated a new application for delivering GM-CSF to alveolar space to protect against different lung diseases. This report extends these findings, clarifies a controversial issue and includes the new innovative aspects of using GM-CSF after influenza infection.

We have provided the first evidence that pulmonary GM-CSF provides extraordinary protection against different IAV strains by stimulating pulmonary innate immunity through alveolar macrophages [2]. We have shown here that intranasal delivery of GM-CSF protects wild type mice with different genotypes, that express two different MHC molecules, C57Bl/6 and Balb/c (Fig 1), through increased proliferation of AMs, production of TNFα, MCP-1 and amphiregulin that is a growth factor, and expression of PU.1 in AMs. This confirms our previous results and the recent findings by Schnider et al on the role of AMs as the main subsets of innate immunity that protects mice against lethal IAV infections upon delivery of GM-CSF to the lungs [2,4], although other subsets of immune cells and macrophages may also play role in GM-CSF protection [29]. It also emphasizes on the role of amphiregulin in influenza infection and confirms our and others reports on the role of amphiregulin in protecting against bacterial co-infection in the lungs [28,30].

One of the controversies is heterologous use of GM-CSF to target the innate immunity of lung [26]. The predicted amino acid sequence of human and murine GM-CSF are 54% identical [21,31,32]. Although the critical disulfide structure is completely conserved between the human and murine growth factors and both have a similar carbohydrate modification pattern, it has been consistently demonstrated that hGM-CSF alone fails to stimulate murine cells and mGM-CSF fails to stimulate human cells [21,33–35]. On the other hand there are a few reports on the stimulatory effect of hGM-CSF only on bone marrow cells of irradiated mice in vivo [17,36,37] and a report on the healing of the wound surface induced by chemotherapy in mice [38].

A recent publication demonstrated that treating mice intranasally with human GM-CSF conferred 80% protection against lethal influenza H1N1 [26]. These results are in stark contrast to the numerous previous publications that have repeatedly established that human and mouse GM-CSF do not cross-react in terms of biological activity and receptor binding [21,33,35,39]. We tested hGM-CSF and mGM-CSF to evaluate their heterologous biological activities in bone marrow proliferation assay in vitro (data not shown), in vivo alveolar macrophages proliferation assays (Fig 2A), production of cytokines (Fig 2B and 2C), and amphiregulin (Fig 2D), expression of PU.1 transcription factor (Fig 2E), and in protecting against influenza virus using two different mouse genotypes (Fig 1). Our analysis clearly demonstrated that human GM-CSF had no cross-reactivity with mouse GM-CSF from the biological activity point of view and that human GM-CSF neither stimulated mouse immune cells nor protected mice against influenza virus. This contradicts with the recent publication by Huang et al [26] and re-confirms previous publications demonstrating that human GM-CSF does not activate murine cells [21,33–35]. One of the possible factors may be the virus strains used in these two experiments. We used a common mouse adapted PR8 strain of influenza virus that is lethal to mice whereas Huang et al used a mouse adapted strain FM1 (H1N1) that has been isolated from a patient in 1947. Although using different virus strains may explain the discrepancy in the outcome of protection studies, but they cannot explain the inability of hGM-CSF in stimulating mouse immune cells (Fig 2A–2E). Hence, we speculate that the use of a different hGM-CSF preparation by Huang et al may have played a role in the observed protection via non-specific inflammation of the lungs, since non-specific lung inflammation has been shown to enhance resistance to influenza [40].

Elderly population is significantly more vulnerable to seasonal influenza infections and approximately 90% of influenza related deaths in the USA occurs among elderly population of 65 years or older [19,41]. On the other hand, the antibody response to influenza vaccination is reduced in the elderly [42–44] and we have shown that T cell responses to influenza is significantly reduced in aged mice [45]. Our results provide a proof of principle for a novel strategy that can be deployed to boost the local innate immunity of the elderly population in the respiratory tract in general and in the lungs in particular. Delivery of GM-CSF to the respiratory tract not only will be helpful for protecting the elderlies against influenza, but also can be used to protect against other community acquired pneumonias.

Our data and previous publication from our labs and others show that delivering GM-CSF to the lungs before infection protects mice against lethal pneumonia due to different strains of IAV (Figs 1 and 3) and [2,4,5,26,28]). GM-CSF will be clinically very useful if given to patients after the onset of influenza. But treating IAV-infected mice with GM-CSF was challenging as IAV-infected mice were more susceptible to GM-CSF treatment particularly if given after 2 days post infection. Hence, we tested the notion that the IAV-induced lung injury may play a pivotal role in mortality of GM-CSF-treated IAV-infected mice. We have already shown that IAV-infected mice have elevated albumin levels in their BAL [2]. Our current report shows that treating IAV-infected mice with GM-CSF at 3 and 6 days after IAV infection will result in dissemination of GM-CSF to the blood circulation (Fig 4A). Also, Qdots of 20nm size delivered intravenously to IAV infected mice were found in abundance in BAL (Fig 4B and 4C). Altogether, this shows that lung injury induced by IAV compromises the integrity of lungs barrier and based on our current protocol in a mouse model, high doses of GM-CSF in alveolar space (~100x of the normal systemic dose) is needed to activate AMs and protect mice against lethal IAV. Hence, preexisting pulmonary diseases, as well as influenza-mediated lung injury in itself, induces a porous lung vasculature/epithelium that facilitates the escape of GM-CSF from the alveolar space, reducing its effectiveness as a therapeutic agent.

The covalent attachment of nanoparticles to therapeutic proteins enhances their bioavailability, prolongs half-lives and reduces immunogenicity [46]. Ten products conjugated to nanoparticles have been FDA-approved, and four are blockbuster drugs [46]: conjugated IFN-α2a, IFN-α2b, G-CSF and epoietin. Conjugated IFN-α2a and IFN-α2b are more effective and less toxic than their plain forms, and are the treatment of choice for hepatitis B and C [47,48]. Conjugating GM-CSF to nanoparticles is feasible, and enhances DCs activity [20]. Mouse recombinant GM-CSF in subcutaneous administration had a distribution half life of 0.92±0.04 minutes and an elimination half-life of 11.75±3.89 minutes, much shorter than PEGylated GM-CSF with distribution half-life of 15.9±1.5 minutes and an elimination half-life of 5.3 hours ±13 minutes [20]. We deployed a different approach and conjugated mGM-CSF to Qdot (625) to increase its size and consequently increase its retention in the alveolar space. When we treated IAV-infected C57Bl/6 wild type mice with either Qdot-mGM-CSF, PBS, or plain mGM-CSF, all three groups displayed comparable morbidity, as evidenced by similar weight loss patterns, indicating similar infection rate. However, whereas all of the mice received Qdot-mGM-CSF survived, 40% of PBS-treated group (2 out of 5), 20% of Qdot only treated group and 29% of plain mGM-CSF-treated group (2 out of 7) succumbed (Fig 5C). This demonstrated that despite the reduced in vitro biological activity (Fig 5), the Qdot-mGM-CSF displayed greater protective efficacy in treating influenza infected mice compared to the plain mGM-CSF. Taken together, these results somewhat confirms our hypothesis that retaining mGM-CSF in the alveolar space is a pragmatic strategy to address IAV-induced lung injury and emphasizes on needs for additional work to optimize this strategy. This will open new avenues for therapeutic use of GM-CSF in IAV and other acute lung infections.
